# Klotho Deficiency Promotes Skeletal Muscle Weakness and Is Associated with Impaired Motor Unit Connectivity †

**DOI:** 10.3390/ijms26167986

**Published:** 2025-08-19

**Authors:** Linda A. Bean, Connor Thomas, Juan F. Villa, Alexander J. Fitt, Areli Jannes S. Javier, Akanksha Agrawal, Hanna Whitney, Guilherme Nascimento Dos Santos, Kenneth E. White, Joshua R. Huot, Steven S. Welc

**Affiliations:** 1 Department of Anatomy, Cell Biology & Physiology, Indiana University School of Medicine, Indianapolis, IN 46202, USA; linbean@iu.edu (L.A.B.); juan.villa@uces.edu.co (J.F.V.); akanagra@iu.edu (A.A.); hanna.whitney@aol.com (H.W.); gdossant@iu.edu (G.N.D.S.); jrhuot@iu.edu (J.R.H.); 2 School of Medicine, Universidad CES, Medellin 050021, Colombia; 3 Indiana Center for Musculoskeletal Health, Indiana University School of Medicine, Indianapolis, IN 46202, USA; 4 Department of Medical and Molecular Genetics, Indiana University School of Medicine, Indianapolis, IN 46202, USA

**Keywords:** Klotho, skeletal muscle, wasting, motor unit

## Abstract

Muscle wasting and weakness are critical clinical problems that limit mobility and independence, reduce health span, and increase the risk of physical disability. The molecular basis for this has not been fully determined. Klotho expression is downregulated in conditions associated with muscle wasting, including aging, chronic kidney disease, and myopathy. The objective of this study was to investigate a mechanistic role for Klotho in regulating muscle wasting and weakness. Body weight, lean mass, muscle mass, and myofiber caliber were reduced in Klotho-deficient mice. In the tibialis anterior muscle of Klotho-null mice, type IIa myofibers were resistant to changes in size, and muscle composition differed with a higher concentration of type IIb fibers to the detriment of type IIx fibers. Glycolytic GPDH enzymatic activity also increased. Klotho-deficient mice showed impaired muscle contractility, with reduced twitch force, torque, and contraction–relaxation rates. RNA sequencing revealed upregulation of synaptic and fetal sarcomeric genes, prompting us to examine muscle innervation. Klotho deficiency led to neuromuscular junction remodeling, myofiber denervation, and functional motor unit loss. Loss of motor units correlated with absolute torque. Collectively, these findings reveal a novel mechanism through which systemic Klotho deficiency disrupts muscle synapses and motor unit connectivity, potentially contributing to muscle wasting and weakness.

## 1. Introduction

The long-term ability of individuals to preserve their physical capacity and independence largely depends on maintaining skeletal muscle mass and function. Muscle wasting contributes to weakness and overall physical decline, increasing the risk of falls, injury, and subsequent disability [1,2,3,4]. Furthermore, systemic metabolic adaptations, like insulin resistance and inflammation, occurring with changes in skeletal muscle function, may act as disease modifiers affecting pathophysiology and prognosis [5]. Muscle wasting and persistent weakness impact quality of life and limit the ability of individuals to perform normal daily activities, to work to earn a living, or to return to work after critical illness, owing to considerable societal burden and health care expenditure [6,7]. With aging being a well-known contributing factor to loss of muscle mass, the costs of muscle weakness and its associated complications will continue to rise dramatically as the aging population rapidly expands in size [8]. While there is considerable evidence to support that muscle mass and function are strong prognostic indicators in various medical conditions, the underlying molecular regulators of muscle mass and function have yet to be fully established.

Mutations to the ‘aging suppressor’ Klotho gene result in a shortened lifespan and changes to numerous tissues resembling premature aging [9]. The Klotho gene encodes a transmembrane protein with a large extracellular component. Klotho expression is restricted to several tissues, including skeletal muscle. However, its function is best known where it is abundantly expressed, in the kidney, acting as a co-receptor for fibroblast growth factor 23 to regulate phosphate and mineral homeostasis [10]. The extracellular fraction can be enzymatically cleaved and released as a soluble factor with pleiotropic, discrete functions, independent of FGF23, including modification of insulin/IGF-1, TGFβ, NF-κB, Wnt, and NRF2 signaling [11].

Klotho may function as a regulator of muscle mass and weakness. Both circulating Klotho levels and muscle mass begin to decline after 40 years of age [12]. Low Klotho correlates with frailty and increased occurrences of falls in nursing home residents [13,14,15]. Plasma Klotho levels are negatively associated with daily living disability and positively associated with lean mass [16,17]. Low Klotho levels also correlate with poor muscle strength and early mortality [18].

In the present study, we tested the effects of Klotho deficiency on muscle mass and function. We assayed for changes in the composition of fast-twitch tibialis anterior (TA) and slow-twitch soleus muscles and distinguished morphology by fiber type. Klotho deficiency resulted in a shift from type IIx toward a higher proportion of type IIb fibers in TA muscles, which was paralleled by increased activity of the glycolytic enzyme α-glycerolphosphate dehydrogenase (GPDH). We identified transcriptional reprogramming associated with altered expression of sarcomeric and synaptic genes. We then extended our hypothesis beyond muscle-level changes to test if Klotho deficiency affects motor unit connectivity, a determinant of muscle function. We used an established motor unit number estimation (MUNE) technique to evaluate the number of motor neurons functionally connected to muscle. Histological assessment further demonstrated enhanced expression of the denervation marker neural cell adhesion molecule (NCAM), reduced area of synaptic contact, and neuromuscular junction (NMJ) morphological alterations. Taken together, these results support an important role for Klotho in the regulation of muscle mass, composition, and contractile and neuromuscular function.

## 2. Results

### 2.1. Genetic Ablation of Klotho Reduces Lean Mass, Fat Mass, and Muscle Mass

**Figure 1:**
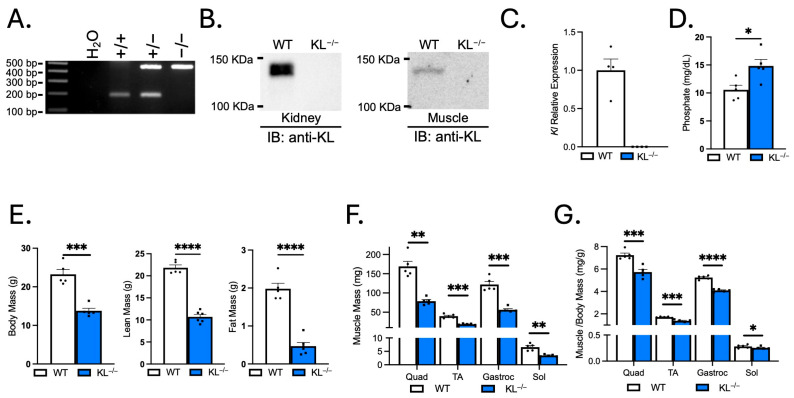
Klotho-deficient mice have reduced body mass, lean mass, fat mass, and muscle mass. (**A**) Representative images of wild-type (186 bp) and mutant (426 bp) PCR products from wild-type, heterozygous, and homozygous mice distinguish genotype. (**B**) Immunoblot confirms no detectable Klotho expression in kidney or muscle lysates of KL^−/−^ mice. (**C**) qRT-PCR assays show *Kl* mRNA expression is not detectable in KL^−/−^ muscle lysates. *n* = 4 per group. (**D**) Serum phosphate levels were characteristically increased in KL^−/−^ mice. *n* = 5 per group. (**E**) Body mass and body composition were assessed by Echo-MRI, showing decreased lean mass and fat mass in KL^−/−^ mice. Muscle mass (**F**) and muscle mass normalized to body mass (**G**) of the quadriceps (quad), tibialis anterior (TA), gastrocnemius (gastroc), and soleus (sol) in wild-type and KL^−/−^ mice. *n* = 5–6 per group. Data are presented as mean ± SEM, with black dots showing individual data points. All *p* values are based on two-tailed *t* tests. * *p* < 0.05, ** *p* < 0.01, *** *p* < 0.001, **** *p* < 0.0001 versus wild-type.

Systemic soluble Klotho concentrations are suppressed with aging and associated with muscle atrophy [9,12]. Klotho levels are also reduced locally in skeletal muscles with aging, acutely in response to muscle injury, and chronically after the onset of inflammation in dystrophic muscles [19,20,21]. The first objective of this study was to examine the effects of systemic Klotho deficiency on skeletal muscle wasting. Mouse genotypes were determined by PCR (Figure 1A), and immunoblots confirmed the absence of Klotho expression in KL^−/−^ kidney and muscle lysates (Figure 1B). Normal muscles expressed full-length (130-kDa) Klotho protein. qRT-PCR also showed that *Kl* mRNA was not detectable in KL^−/−^ muscles (Figure 1C). As expected, serum phosphate levels were elevated in KL^−/−^ mice (WT: 10.6 ± 0.8 vs. KL^−/−^: 14.8 ± 1.2 mg/dL, *p* < 0.05, Figure 1D).

Klotho deficiency resulted in a ~40% decrease in body mass (*p* < 0.001, Figure 1E). Body composition assessment revealed a reduction in lean (−51%, *p* < 0.0001) and fat mass (−76%, *p* < 0.0001, Figure 1E). The gross wet mass of quadriceps (−53%, *p* < 0.01), TA (−52%, *p* < 0.001), gastrocnemius (−54%, *p* < 0.001), and soleus muscles (−48%, *p* < 0.01) was reduced in KL^−/−^ mice (Figure 1F). The muscle mass to body mass ratio was also reduced in the quadriceps (−21%, *p* < 0.001), TA (−19%, *p* < 0.001), gastrocnemius (−22%, *p* < 0.0001), and soleus muscles (−11%, *p* < 0.05), indicating that effects on muscle mass exceeded allometric scaling (Figure 1G).

### 2.2. Klotho Deficiency Reduces Myofiber Size and Affects Muscle Composition in Fast-Twitch TA Muscles

**Figure 2:**
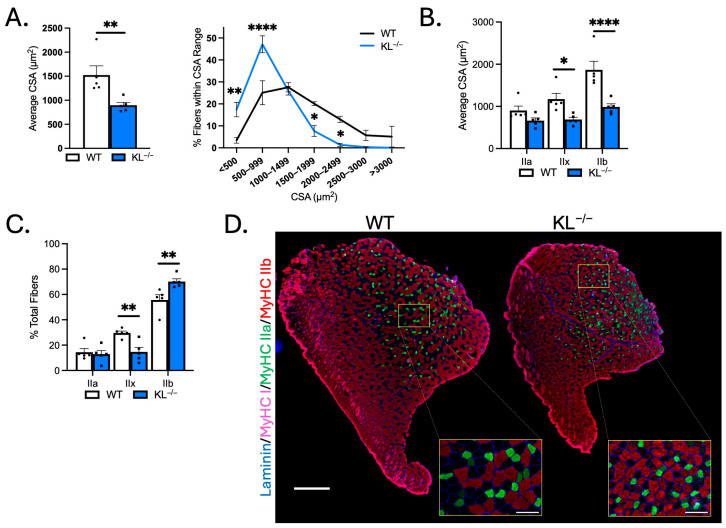
Genetic Klotho deficiency reduces muscle fiber size and affects fiber-type composition in TA muscles. (**A**) (**left**) Mean muscle fiber cross-sectional area (CSA). *n* = 5 per group. *p* values based on two-tailed *t* tests. (**right**) Frequency distribution of muscle fiber size by CSA. (**B**) Quantification of muscle fiber CSA by fiber type in TA muscles from wild-type and KL^−/−^ mice. (**C**) Quantification of TA muscle composition by fiber type. *n* = 5 per group. (**D**) Representative montages of whole TA cross-sections from wild-type (**left**) and KL^−/−^ (**right**) mice immunolabeled with antibodies to laminin (blue), myosin heavy chain type (MyHC) I (magenta), MyHC IIa (green), MyHC IIb (red), and MyHC IIx unlabeled (black). Montage bar = 500 μm. Inset bar = 100 μm. *n* = 5 per group. Data are presented as mean ± SEM, with black dots showing individual data points. Unless otherwise indicated, *p* values are based on two-way ANOVA with the Šidák multiple comparison test (B-D, G-I). * *p* < 0.05, ** *p* < 0.01, **** *p* < 0.0001 versus wild-type.

Myofiber CSA was assayed in the mid-belly of muscles to determine if reductions in muscle mass are due to differences in size. Mean myofiber size was reduced in TA muscles of KL^−/−^ mice (−41%, *p* < 0.01), marked by an increase in the proportion of small fibers (≤999 µm^2^) and a reduction in the percentage of large fibers (1500–2499 µm^2^; Figure 2A). Next, we evaluated changes in CSA by fiber type on sections immunolabeled with antibodies to MyHC types I, IIa, and IIb. Type IIx fibers were identified by the absence of staining. The CSAs of type IIx (−41%, *p* < 0.05) and type IIb fibers (−47%, *p* < 0.0001) were reduced (Figure 2B,D). Whereas no significant change in type IIa fiber size suggests fiber-type-related resistance to the effects of Klotho deficiency. Muscle composition was altered with increased frequency of type IIb fibers (WT: 55.7% vs. KL^−/−^: 70.1%, *p* < 0.01) at the expense of type IIx fibers (WT: 29.6% vs. KL^−/−^: 14.6%, *p* < 0.01, Figure 2C,D).

### 2.3. Klotho Deficiency Reduces Myofiber Size but Does Not Affect Muscle Composition in Slow-Twitch Soleus Muscles

**Figure 3:**
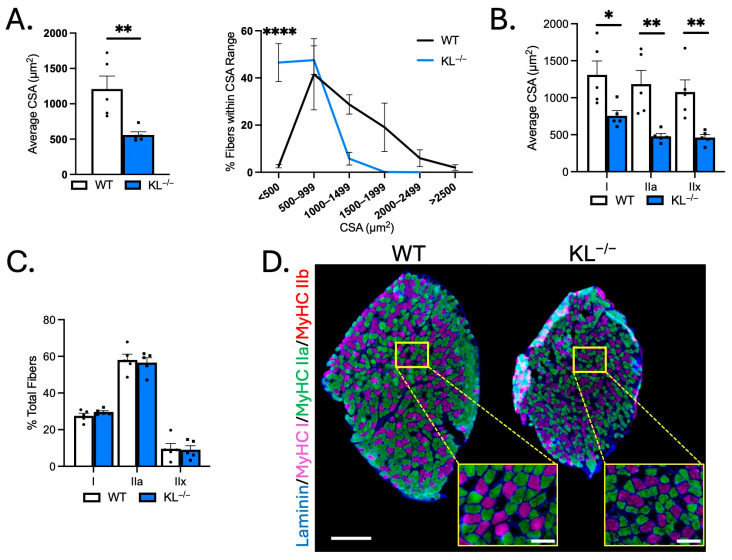
Genetic Klotho deficiency reduces muscle fiber size in soleus muscles. (**A**) (**left**) Mean muscle fiber cross-sectional area (CSA). *n* = 5 per group. *p* values based on two-tailed *t* tests. (**right**) Frequency distribution of muscle fiber size by CSA. (**B**) Quantification of muscle fiber CSA by fiber type in soleus muscles from wild-type and KL^−/−^ mice. *n* = 5 per group. (**C**) Quantification of soleus muscle composition by fiber type. *n* = 5 per group. (**D**) Representative montages of whole soleus muscle cross-sections from wild-type (**left**) and KL^−/−^ (**right**) mice immunolabeled with antibodies to laminin (blue), myosin heavy chain type (MyHC) I (magenta), MyHC IIa (green), MyHC IIb (red), and MyHC IIx unlabeled (black). Montage bar = 250 μm. Inset bar = 50 μm. Data are presented as mean ± SEM, with black dots showing individual data points. *p* values are based on two-way ANOVA with the Šidák multiple comparison test (B-D, G-I). * *p* < 0.05, ** *p* < 0.01, **** *p* < 0.0001 versus wild-type.

To test for differential responses of slow-twitch muscles to Klotho deficiency, fiber-type and morphological analyses were performed on soleus. TA muscles are comprised primarily of type IIx and IIb fast/glycolytic fibers, and the soleus consists of mostly type I and IIa slow/oxidative fibers. Mean CSA of soleus myofibers was reduced in KL^−/−^ mice (−53%, *p* < 0.01) and associated with an increased frequency of small myofibers (<500 µm^2^, *p* < 0.0001, Figure 3A). CSA was reduced in types I (−42%, *p* < 0.05), IIa (−60%, *p* < 0.01), and IIx fibers (−57%, *p* < 0.01, Figure 3B,C) indicating that fast/glycolytic and slow/oxidative fiber types are susceptible to the effects of Klotho deficiency in soleus. No difference in soleus composition was observed (Figure 3C,D). These data indicate distinct effects of Klotho deficiency on muscle composition and type IIa myofiber size between TA and soleus muscles.

### 2.4. Genetic Ablation of Klotho Increases Glycolytic Metabolic Activity in TA Muscles

**Figure 4:**
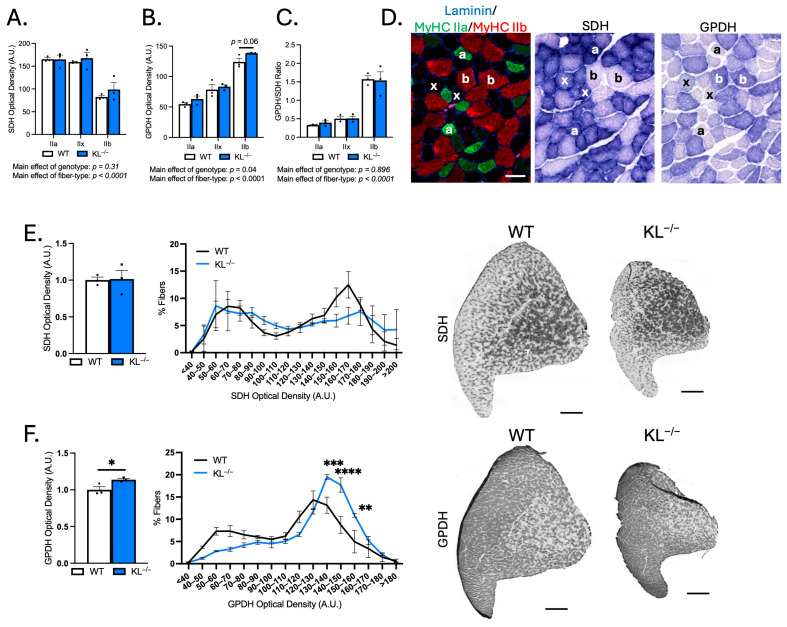
Genetic ablation of Klotho increases glycolytic GPDH enzymatic activity in TA muscles. (**A**–**C**) Serial cryosections of TA muscles immunolabeled for MyHC isoforms or succinate dehydrogenase (SDH) or glycerol-3-phosphate-dehydrogenase (GPDH) enzyme histochemistry. Quantification of SDH (**A**) and GPDH (**B**) optical density and GPDH-to-SDH ratio (**C**) by fiber type. *n* = 3 per group. *p* values based on two-way ANOVA with Tukey’s multiple comparison test. (**D**) Representative images of the same region of TA cross-sections. (**left**) Cross-sections immunolabeled for laminin (blue), MyHC I (magenta), MyHC IIa (green), MyHC IIb (red), and MyHC IIx unlabeled (black). (**middle**) SDH enzyme histochemistry. (**right**) GPDH enzyme histochemistry. Symbols indicate the following: a = type IIa, x = type IIx, and b = type IIb fiber types across images. Bar = 50 μm. (**E**) (**left**) Mean SDH enzyme activity. *p* values based on two-tailed *t* tests. (**middle**) Frequency distribution of SDH activity independent of fiber type. (**right**) Representative images of SDH activity in whole TA montages from wild-type and KL^−/−^ mice in gray-scale. Montage bar = 500 μm. (**F**) (**left**) Mean GPDH enzyme activity. *p* values based on two-tailed *t* tests. (**middle**) Frequency distribution of GPDH activity independent of fiber type. Data are presented as mean ± SEM, with black dots showing individual data points. *p* values based on two-way ANOVA with the Šidák multiple comparison test. (**right**) Representative images of GPDH enzyme histochemistry of whole TA cross-section montages from wild-type and KL^−/−^ mice in gray-scale. Montage bar = 500 μm. *n* = 3 per group. * *p* < 0.05, ** *p* < 0.01, *** *p* < 0.001, **** *p* < 0.0001 versus wild-type.

Because muscle fiber-type switching can affect the metabolic properties of muscle, we performed histochemical analysis on TA muscles to determine if Klotho deficiency affects the aerobic-oxidative and anaerobic-glycolytic activities. Serial sections were used for identification of fiber types by immunofluorescence or for histochemical analysis of SDH and GPDH activities (Figure 4A–D). As expected, SDH activity was highest in type IIa fibers, and GPDH activity and the GPDH-to-SDH ratio were highest in type IIb fibers. No change in SDH activity was detected, but we observed a significant main effect of genotype on GPDH activity in KL^−/−^ muscles (*p* < 0.05, Figure 4B). Despite increased GPDH activity, the GPDH-to-SDH ratio remained unchanged (Figure 4C), suggesting that the metabolic alterations may reflect global remodeling rather than a classic shift from oxidative to glycolytic metabolism associated with fiber-type transitions. Next, we expanded our analyses to all fibers of TA muscles, independent of sub-type (Figure 4E,F). No changes in mean and SDH activity distribution were observed (Figure 4E). However, consistent with a higher proportion of type IIb fibers, mean GPDH activity increased (+14%, *p* < 0.05), and we noted a higher frequency of fibers with high GDPH activity (Figure 4F). These findings show that changes in muscle fiber-type composition are coupled with a corresponding shift towards increased glycolytic metabolic activity in TA muscles from Klotho-deficient mice.

### 2.5. Increased Transcriptional Expression of Sarcomeric and Synaptic Genes in Klotho-Deficient TA Muscles

**Figure 5:**
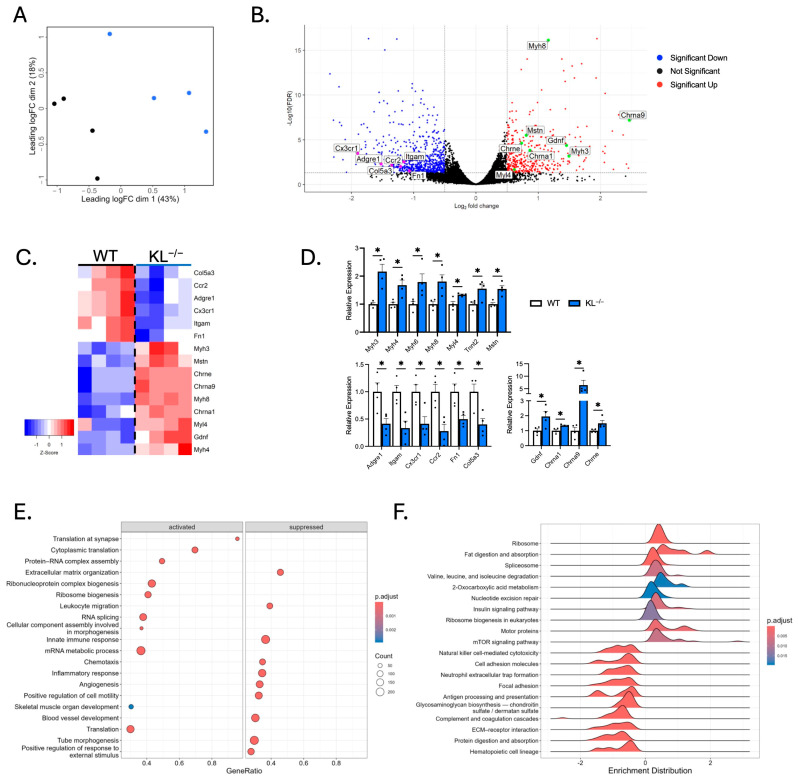
RNA sequencing analysis shows distinct gene expression profiles in skeletal muscles from Klotho-deficient mice. (**A**) Multi-dimensional scaling plot comparing TA muscles from wild-type and KL^−/−^ mice. *n* = 4 per group. Black = wild-type. Blue = KL^−/−^. (**B**) A volcano plot highlighting selected differentially expressed genes (DEGs). Increased expression (red) and decreased expression (blue) in KL^−/−^ muscles. Colored dots = abs(log_2_0.5) fold change with FDR < 0.05. (**C**) A heatmap of samples showing select DEGs in WT and KL^−/−^ muscles. Positive (red) and negative (blue) Z-scores. (**D**) qRT-PCR assays validating differential expression of selected sarcomeric (**top**), inflammatory and pro-fibrotic (**bottom left**), and acetylcholine receptor and neurotrophic genes (**bottom right**). *n* = 4 per group. Data are presented as mean ± SEM, with black dots showing individual data points. *p* values based on two-tailed *t* tests. * *p* < 0.05 versus wild-type. (**E**) Dot plot depicting activated (**left**) and suppressed (**right**) GO of Biological Process terms in KL^−/−^ muscles. (**F**) Ridgeline plot, grouped by gene set, representing activated and suppressed KEGG pathways in KL^−/−^ muscles.

To assess the molecular mechanisms behind muscle wasting, composition, and metabolic activities observed with Klotho deficiency, we performed RNA sequencing analysis. Multi-dimensional scaling showed distinct separation of WT and KL^−/−^ TA muscles (Figure 5A). There were 2124 differentially expressed genes utilizing a false discovery rate <0.05 threshold; 1046 of those differed by at least log_2_ 0.5-fold, and of those, 721 genes were upregulated and 392 downregulated (Figure 5B). Candidate genes were examined for expressional changes (Figure 5C). We observed increased expression of *Mstn,* a negative regulator of muscle growth, but no change in the expression of pro-atrophy genes *Fbxo32* or *Trim63*. The expression of sarcomeric transcripts *Myh3*, *Myh4*, *Myh6*, *Myh7b*, *Myh8*, *Myh13*, *Myl4*, *Myl10*, *Tnnc1*, *Tnni1*, and *Tnnt2* was elevated in KL^−/−^ muscle. Increased *Myh4* expression was consistent with a higher concentration of type IIb myofibers (Figure 2C). *Myh3*, *Myh8*, *Myl4*, and *Tnnt2* genes are expressed highest during development; their expression is downregulated postnatally but re-expressed in response to muscle injury or denervation [22,23,24]. In this regard, transcripts for acetylcholine receptors *Chrna1*, *Chrna9*, and *Chrne* and motor neuron survival factor *Gdnf* were elevated in the KL^−/−^ muscle. However, we did not observe transcriptional changes associated with muscle damage. No change in expression of myogenic factors *Pax7*, *Myod1*, *Myog*, *Myf5*, and *Myf6*, and reduced expression of inflammatory and extracellular remodeling genes, including macrophage markers *Adgre1* and *Itgam*, chemokine receptors *Cx3cr1* and *Ccr2*, and extracellular matrix components *Fn1* and *Col5a3*. Expressional changes of key transcripts were validated by qRT-PCR (Figure 5D).

To identify biological processes in muscle affected by Klotho deficiency, GSEA and GO analyses were performed. We found positive enrichment for translation at the synapse, cytoplasmic translation, protein–RNA complex assembly, ribosome biogenesis, and skeletal muscle development in KL^−/−^ muscle (Figure 5E). In contrast, extracellular matrix organization, leukocyte migration, and innate immune response were downregulated terms. KEGG pathway enrichment analysis was also performed to gain mechanistic insights (Figure 5F); the top enriched pathways include ribosome and spliceosome processes, which play essential roles in normal cell physiology by regulating protein synthesis and gene expression. Other enriched pathways include increased valine, leucine, and isoleucine degradation and 2-oxocarboxylic acid metabolism. In accord with Klotho repressing insulin and IGF-1 signaling [25], enrichment of insulin signaling and mTOR signaling was observed in KL^−/−^ muscles. Interestingly, mTOR signaling is a primary driver of muscle wasting and NMJ dysfunction [26,27]. Collectively, these data raise the possibility that Klotho deficiency facilitates changes to the muscle synapse, promoting muscle wasting and altered expression of sarcomeric transcripts.

### 2.6. Klotho Deficiency Promotes Muscle Weakness

**Figure 6:**
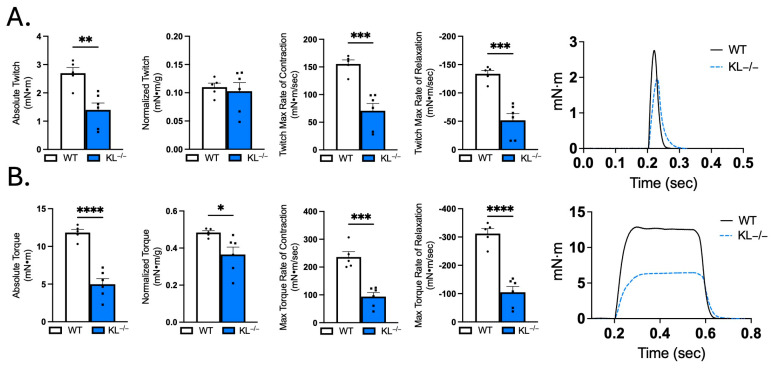
Muscle contractile function is impaired in Klotho-deficient mice. In vivo plantar flexion force assessment of the triceps surae muscle group in WT and KL^−/−^ mice is reported as follows: (**A**) absolute twitch (mN·m), twitch normalized to body weight (mN·m per g body weight), twitch max rate of contraction and relaxation, and representative max twitch trace; (**B**) absolute torque (mN·m), tetanic torque normalized to body weight (mN·m per g body weight), max torque rate of contraction and relaxation, and representative max torque trace. *n* = 5–6 per group. Data are presented as mean ± SEM, with black dots showing individual data points. All *p* values are based on two-tailed *t* tests. * *p* < 0.05, ** *p* < 0.01, *** *p* < 0.001, **** *p* < 0.0001 versus wild-type.

The observation that Klotho deficiency affects myofiber size and composition indicates that Klotho may affect muscle function. We tested that possibility by performing in vivo plantarflexion functional analysis. Absolute muscle twitch was 48% lower, indicating reduced force to a single impulse in KL^−/−^ mice (*p* < 0.01, Figure 6A). However, there was no effect on muscle twitch scaled to body weight (Figure 6A). Muscle twitch maximal rates of contraction (−54%, *p* < 0.001) and relaxation (−61%, *p* < 0.001, Figure 6A) were reduced in KL^−/−^ mice. Reductions in absolute torque (−58%, *p* < 0.0001) and tetanic torque normalized to body weight (−23%, *p* < 0.05, Figure 6B) indicate muscle weakness independent of differences in body mass in KL^−/−^ mice. Maximal rate of torque development (−60%, *p* < 0.001) and relaxation (−66%, *p* < 0.0001, Figure 6B) also decreased in KL^−/−^ mice. Taken together, Klotho deficiency promotes muscle weakness and alters muscle contraction and relaxation rates.

### 2.7. Motor Unit Connectivity Is Impaired in Klotho-Deficient Mice

**Figure 7:**
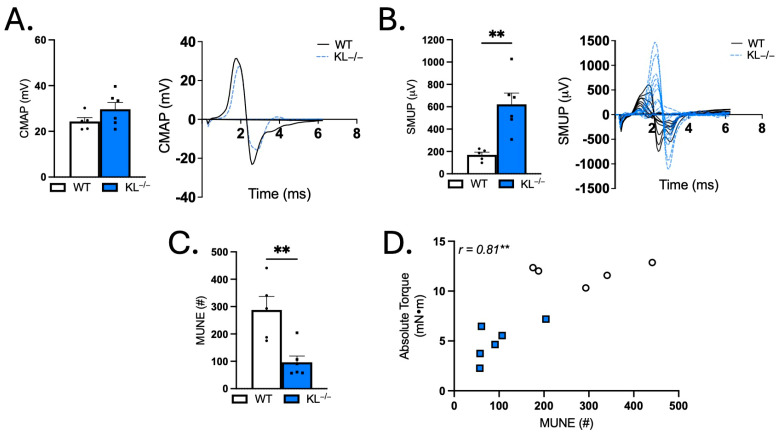
Genetic Klotho deficiency alters single motor unit potential (SMUP) and motor unit number estimation (MUNE). (**A**) Compound muscle action potential (CMAP; millivolts (mV)). Representative trace of CMAP in WT and KL^−/−^. (**B**) SMUP (microvolts (μV)). Representative incremental traces of SMUP in WT and KL^−/−^. (**C**) MUNE (number of motor units (#)). (**D**) In vivo plantar flexion absolute torque correlated with MUNE. *n* = 5–6 per group. Data are presented as mean ± SEM, with black dots showing individual data points. All *p* values are based on two-tailed t tests (**A**–**C**) or correlation analysis for Pearson r values. ** *p* < 0.01 versus wild-type.

Given that Klotho deficiency promotes weakness, alters contraction and relaxation dynamics, and transcriptional analysis shows changes associated with the muscle synapse, we next sought to test indices of motor unit connectivity. Electrophysiological measurements showed no change in baseline-to-peak assessment of CMAP (Figure 7A). However, SMUP increased 3.6-fold in KL^−/−^ mice relative to WT (*p* < 0.01, Figure 7B). MUNE calculations showed a robust 67% reduction, indicating reduced motor units with Klotho deficiency (*p* < 0.01, Figure 7C). Correlation analysis revealed that MUNE is positively associated with absolute torque (Pearson r = 0.81, *p* < 0.01, Figure 7D). These data indicate that impaired motor unit connectivity is associated with muscle weakness in Klotho-deficient mice.

### 2.8. Klotho Deficiency Alters the Structure and Synaptic Overlap at the NMJ of TA Muscles

**Figure 8:**
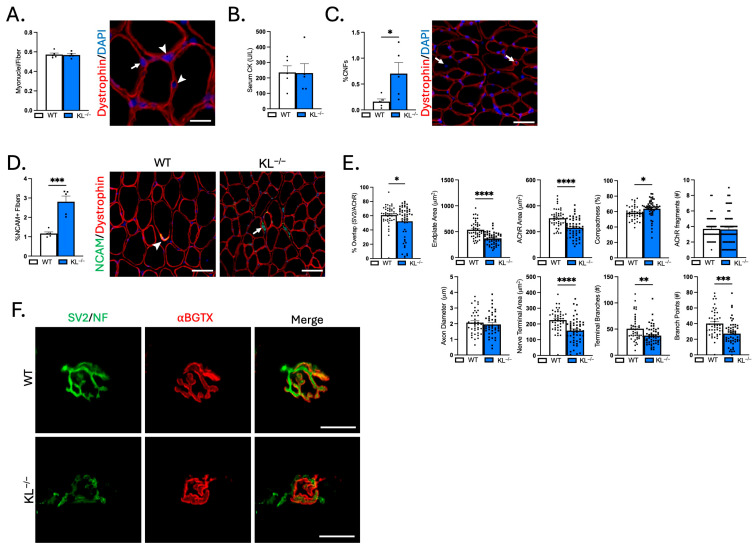
Genetic ablation of Klotho increases the concentration of denervated muscle fibers, reduces synaptic contact, and alters NMJ morphology in TA muscles. (**A**) Myonuclei number per muscle fiber. *n* = 5 per group. Representative image of a muscle cross-section immunolabeled with dystrophin and stained with DAPI to visualize nuclei for quantification of myonuclei. Arrows = myonuclei. Arrowheads = interstitial nuclei. Bar = 20 μm. (**B**) Serum creatine kinase (CK) levels. *n* = 5 per group. (**C**) Quantification of the percentage of muscle fibers with centrally located nuclei to total muscle fibers. *n* = 5 per group. Representative image of a muscle cross-section immunolabeled with dystrophin and stained with DAPI to quantify centrally nucleated fibers (CNFs). Arrow = CNF. Bar = 50 μm. (**D**) Quantification of the proportion of denervated muscle fibers expressing NCAM. *n* = 5 per group. Representative images of muscle sections immunolabeled with dystrophin (red) and NCAM (green) from wild-type (**left**) and KL^−/−^ mice (**right**). Arrowhead = NMJ with high expression of NCAM. Arrow = small angulated denervated fiber with high cytosolic expression of NCAM. Bar = 50 μm. (**E**) Quantification of NMJ morphological properties of longitudinal sections of TA muscles from wild-type and KL^−/−^ mice. Percent co-localization of synaptic vesicles and AChRs; post-synaptic NMJ morphology endplate area, AChR area, compactness (AChR area/endplate area), and number of AChR fragments; and pre-synaptic NMJ morphology axon diameter, nerve terminal area, number of terminal branches, and branch points. In dispersion plots, each point represents a single NMJ from *n* = 5 wild-types and *n* = 6 KL^−/−^ biological replicates, with *n* = 6–15 *en face* NMJs quantified per biological replicate. (**F**) Representative images of longitudinally cut TA muscle sections from wild-type (top) and KL^−/−^ mice (bottom) stained with bungarotoxin (red) and immunolabeled with SV2 and neurofilament (green). Bar = 20 μm. Data are presented as mean ± SEM, with black dots showing individual data points. All *p* values are based on two-tailed *t* tests. * *p* < 0.05, ** *p* < 0.01, *** *p* < 0.001, **** *p* < 0.0001 versus wild-type.

Muscle wasting, weakness, altered composition, and functional loss in motor unit innervation could result from changes to the NMJ. First, we quantified the number of myonuclei per fiber to test for effects on myonuclear accretion. No change indicates that reduced myofiber size was not associated with effects to the myonuclear domain (Figure 8A). To address the effects of Klotho deficiency on muscle injury, serum CK was analyzed and the proportion of centrally nucleated fibers (CNFs) was quantified. Serum CK levels did not change with Klotho deficiency (Figure 8B). However, there was a small but significant increase in CNFs (WT: 0.2% vs. KL^−/−^: 0.7%, *p* < 0.05, Figure 8C). Central nucleation is a well-recognized indicator of regeneration, but changes in nuclear placement may also occur with denervation [28,29]. To address this point, we assessed myofiber expression of NCAM, whose expression accumulates intracellularly in denervated fibers [30]. Indeed, the percentage of NCAM+ fibers increased in KL^−/−^ muscles. NCAM was most prevalent in small, angulated fibers consistent with denervation (*p* < 0.0001, Figure 8D).

To gain insight into whether Klotho deficiency affects NMJ structure, morphological analysis was performed on longitudinal sections stained with antibodies to neurofilament to identify the axon, SV2 to label the nerve terminal, and α-bungarotoxin to visualize post-synaptic AChRs (Figure 8E,F). Overlap between pre-synaptic and post-synaptic components decreased, indicating reduced area of synaptic contact in KL^−/−^ mice (−14%, *p* < 0.05, Figure 8E). Pre- and post-synaptic morphology were also altered with Klotho deficiency (Figure 8F). Endplate area (−32%, *p* < 0.0001) and AChR area (−24%, *p* < 0.0001) decreased, whereas compactness of AChRs at the endplate increased (+9%, *p* < 0.05, Figure 8E). At the pre-synapse, the nerve terminal area (−30%, *p* < 0.0001), the number of terminal branches (−26%, *p* < 0.01), and branch points (−32%, *p* < 0.001, Figure 8E) decreased in KL^−/−^ mice. Taken together, these findings demonstrate that the functional loss of motor unit connectivity is associated with increased prevalence of NCAM+ denervated fibers, reduced area of synaptic contact, and morphological changes to the NMJ potentially affecting signal transmission and muscle function.

## 3. Discussion

The major findings of the current study are that Klotho affects muscle mass, composition, function, and motor unit connectivity. These conclusions are based on the following experimentation using Klotho-deficient mice. First, we demonstrated reductions in body weight, lean mass, muscle mass, and myofiber CSA. Second, we showed altered muscle composition with an increased proportion of type IIb fibers and elevated glycolytic GPDH activity in KL^−/−^ muscles. Transcriptomic data revealed positive enrichment for biological processes related to translation at the synapse, as well as increased expression of genes encoding AChRs and sarcomeric genes that are re-expressed in mature muscles after denervation. Finally, we showed that transcriptional changes were coupled with functional loss of motor unit number, increased prevalence of denervated fibers, reduced area of synaptic contact, and small, compact NMJ morphology. Decreased muscle torque also correlated with reductions in motor unit number.

We were very interested to learn that Klotho deficiency resulted in transcriptional, morphological, and functional changes impacting motor unit connectivity. To our knowledge, this is the first report to demonstrate changes to the NMJ and muscle denervation related to Klotho deficiency. However, excess Klotho promotes motor neuron survival [31]. Altered motor nerve function, muscle loss, and weakness are common pathologies observed in clinical conditions of Klotho insufficiency [32,33,34]. Functional consequences related to the loss of neural input in muscle include diminished absolute torque, lower rates of force development and relaxation, and fatigue that manifest as a reduction in strength and coordination resulting in increased susceptibility to falls [35]. Taken together, our findings support that changes to the muscle synapse affecting motor unit connectivity and muscle function may contribute to increased risk of falls and frailty observed in clinical populations with low Klotho levels [13,14,15].

We also observed large reductions in muscle mass and myofiber caliber with Klotho deficiency. Muscles are comprised of fibers classified by sub-type based on fatigability, contractile, and metabolic properties. Considering that different muscles and fiber types within a muscle may respond differently to the same stimulus, we were interested to learn that TA muscles of Klotho-deficient mice had smaller type IIx and IIb but not type IIa fibers. In contrast, all fiber types were smaller in the soleus. Like our observations, type IIa fibers of the TA are resistant to denervation-induced atrophy with aging and neurodegenerative disease [36,37]. However, in denervated soleus muscles, both type I and type II fibers atrophy [38].

Another intriguing observation is that Klotho deficiency altered the composition of the TA, but not the soleus. TA muscles had a higher percentage of type IIb fibers to the detriment of type IIx fibers. Fiber type is a crucial determinant of contractile function and metabolism. Type IIb fibers rely on glycolysis for energy production. Consistent with this, GPDH enzymatic activity increased in TA muscles from Klotho-deficient mice. Neural input directly influences fiber type; a slow-to-fast fiber-type switch occurs when slow fibers are re-innervated by fast motor neurons [39]. Type II fiber dominance is mostly associated with disuse, denervation, and loss of oxidative gene expression [40,41].

The mechanisms underlying muscle wasting in Klotho-deficient mice are multiple. Klotho affects several signaling pathways known to regulate muscle mass [42,43,44], we used RNA sequencing for unbiased characterization of transcriptional changes to muscles. Surprisingly, expression of key regulators of muscle atrophy *Fbxo32* and *Trim63* were not differentially expressed, but *Mstn* expression was elevated. Antithetically, GO analysis showed activation of pathways opposed to catabolism, including protein translation and ribosome biogenesis. One potential explanation for this is that protein synthesis rates of individual fibers increase to mitigate atrophy after denervation [45]. However, others reported increased expression of *Fbxo32* and *Trim63* in the gastrocnemius of Klotho-deficient mice [43]. These discrepancies may reflect the use of distinct muscles or older animals than those examined here. An alternative explanation is a possible uncoupling between transcriptional signaling activation and functional outcomes. Impaired ribosome biogenesis, mitochondrial dysfunction, and endoplasmic reticulum stress can override growth signals in certain contexts, including aging [46,47].

We did not observe an impaired myogenic response in normal muscles of Klotho-deficient mice. Muscle progenitor cells play a crucial role in postnatal growth, fusing to fibers to add nuclei to accommodate increased synthetic demand [48]. In our study, we found no change to the number of myonuclei per fiber. Therefore, muscle wasting does not appear to be related to differences in myonuclear accretion. A role for Klotho in the regulation of muscle progenitor cell function affecting regeneration in response to injury is well established [19,20,21,49,50]. However, serum CK levels were normal, fewer than 1% of myofibers contained central nuclei, and transcriptional expression of key regulators of myogenesis was normal in Klotho-deficient mice. Collectively, these data suggest reductions in muscle mass are not related to impaired myogenesis or injury. An important next step in future investigations will be to use models for the inducible deletion of Klotho to further distinguish effects of Klotho deficiency on adult, mature muscles.

The specific mechanisms through which Klotho deficiency alters the structure and function of the NMJ, affecting innervation and wasting, are not yet known. We used RNA sequencing and pathway analysis to attempt to identify a mechanism through which Klotho deficiency affects muscle innervation. Although bioinformatic inquiry cannot prove a causal mechanism, the data identified activation of insulin and mTOR signaling pathways and activation of associated biological processes downstream, like ribosome biogenesis. Klotho mutant mice have increased insulin sensitivity [51]. mTOR coordinates cellular proliferation, growth, and survival with environmental factors, including nutrient availability and growth factors controlling muscle mass [52]. Furthermore, insulin and IGF-1 signaling enhances activation of mTOR signaling [53]. Emerging evidence supports a key role for mTOR in regulating maintenance of the post-synaptic endplate to preserve NMJ integrity [27,54,55]. Loss of motor unit connectivity may precede declines in muscle mass and contractility and therefore causally contribute to muscle wasting and weakness [56]. This indicates that aberrant activation of insulin and mTOR signaling pathways with Klotho deficiency may alter NMJ morphology by reducing motor unit connectivity, affecting muscle mass and function. However, further studies are needed to examine the temporal progression of muscle weakness and disruption of motor unit integrity to determine if the effects of Klotho deficiency on the muscle synapse are primary drivers or secondary consequences of muscle degeneration. Future work is also warranted to determine how Klotho deficiency affects neural pathways that regulate synaptic integrity and function.

Although our findings show profound effects of Klotho deficiency on skeletal muscle, we are unable to conclude the contribution of muscle Klotho expression to these effects. The function of skeletal muscle Klotho expression is largely unknown. Here, we confirmed ~130 kDa Klotho protein expression in muscle homogenates, and skeletal muscles express FGF receptors 1–4; however, its effects on muscle do not appear to be directed by FGF23 [57]. Prior works showed that transcriptional expression of Klotho in muscles is suppressed transiently after injury [21], with aging [19], and after disease onset in Duchenne muscular dystrophy, a muscle wasting disorder [20]. In support of a role for muscle-derived Klotho, muscle-targeted knockdown by RNA interference was shown to inhibit myogenesis, impairing regeneration after injury [49]. Whereas other works attribute effects of Klotho deficiency to indirect effects driven by phosphate retention and iron accumulation [43,44]. Also plausible are combinatorial effects through which reduced muscle Klotho expression impacts the muscle’s capacity to respond to endocrine or metabolic stress. To this end, in the absence of the influence of endocrine factors, knockdown of Klotho in normal primary muscle cell cultures *in vitro* induced cellular senescence, mitochondrial dysfunction, and altered bioenergetics [49]. Muscle cells that acquire a senescent phenotype accelerate wasting and dysfunction [58].

In summary, clinical observations suggest that reduced circulating Klotho levels correlate with muscle weakness, frailty, increased risk of falls, and reduced ability to perform activities of daily living. Therefore, Klotho may play an important role in the pathogenesis of muscle wasting and weakness. Our findings identify a previously unrecognized role for Klotho in regulating the structure and function of the NMJ, affecting motor unit connectivity. Mechanistically, our data support that Klotho deficiency causes muscle wasting and weakness, at least in part due to loss of neuromuscular integrity. Supporting this conclusion is a strong correlation between motor unit number and muscle torque. Together, these results advance our understanding of the relationship between Klotho, muscle wasting, and weakness.

## 4. Methods

### 4.1. Mice

Klotho^+/−^ mice provided on a mixed background were obtained from Taconic Biosciences (#TF0361, Germantown, NY, USA) and bred to generate wild-type (WT) and homozygous (KL^−/−^) mice. Genomic DNA was extracted from tail snips or ear punches using hot sodium hydroxide and tris [59]. Genotype was determined by PCR reaction (M8296, Promega, Madison, WI, USA) and primers for Klotho mutant (F: 5′-ATGCTCCAGACATTCTCAGC-3′ and R: 5′-GCAGCGCATCGCCTTCTATC-3′) and control products (F: 5′-GATGGGGTCGACGTCA-3′ and R: 5′-TAAAGGAGGAAAGCCATTGTC-3′) separated by agarose gel electrophoresis. Assays were validated in-house. Weaned mice were housed 2–5 per cage in static cages containing environmental enrichment in a specific pathogen-free facility maintained at 21–22 °C with a 12 h light/dark cycle with a chow diet (2018SX, Teklad, St. Louis, MO, USA) and water provided ad libitum. Body composition (EchoMRI™-700, Houston, TX, USA), in vivo plantarflexion torque, and motor unit connectivity were assessed in 45–50-day-old male mice prior to the effects on mortality [60]. Male mice were used to align with our broader studies on Klotho in muscular dystrophy, a disease that primarily affects males. At the time of euthanasia, muscles were harvested, weighed, and frozen in liquid nitrogen or embedded in OCT compound and frozen in liquid nitrogen-cooled isopentane.

### 4.2. Immunofluorescence and Morphological Analysis

Frozen 10 μm transverse and 20 μm longitudinal muscle sections were blocked in PBS with 0.1% Tween 20 containing 5–10% normal donkey serum. Sections were immunolabeled as performed previously [61]. Antibodies used were laminin (1:200; #L9393) and NCAM (1:250; #AB5032) from MilliporeSigma (Burlington, MA, USA); myosin heavy chain (MyHC) I (1.2 μg/mL; #BA-D5), IIa (1.3 μg/mL; #SC-71), IIb (1.9 μg/mL; #BF-F3), dystrophin (2 μg/mL; #Mandys8 (8H11)), synaptic vesicles (0.54 μg/mL; #SV2), and neurofilament (0.46 μg/mL; #2H3) from Developmental Studies Hybridoma Bank (Iowa City, IA, USA). Sections were then probed with fluorochrome-conjugated secondary antibodies anti-mouse IgG2b (#A21242), IgG1 (#A21121), IgM (#A21044), and anti-rabbit IgG (#A11046) from Invitrogen (Carlsbad, CA, USA); or anti-mouse IgG (#715-585-151) and anti-rabbit IgG (#711-545-152) from Jackson ImmunoResearch (West Grove, PA, USA). Acetylcholine receptors (AChR) were labeled with α-bungarotoxin (1:500; #0007, Biotium, Fremont, CA, USA) and nuclei with DAPI. Sections were imaged using a Zeiss AxioObserver 7 microscope, as previously described [62].

Morphological analysis was performed by segmenting myofibers from single-channel fluorescence images of laminin-stained sections using Cellpose (v2.2.3) [63]. Images from Cellpose segmentation were created in ImageJ (v2.1.0/1.53c), and Regions of Interest (ROIs) were eroded with a fixed number of pixels using the plugin LabelsToRois. The resulting ROIs were applied to the original multi-color image for quantification of myofiber cross-sectional area (CSA) and fiber type. Segmentation in Cellpose and myofiber cross-sectional area analysis was confirmed through manual validation. CSA was evaluated on all muscle fibers except for those affected by processing artifacts. Myonuclear number and central nucleation were quantified from dystrophin-labeled sections stained with DAPI. For quantification of NMJ morphology, images were acquired in a z-series at 0.5 µm intervals from longitudinal sections of TA muscles; raw stacks were deconvolved using the nearest-neighbor algorithm and then collapsed using the extended depth-of-focus module. Individual NMJ inset images were duped out of the full image, then split into separate channels; the red and green channels were merged to create a 2-color, 2-channel hyperstack for processing using an ImageJ-based macro NMJ-morph [64].

### 4.3. Histochemistry

To assess oxidative capacity, succinate dehydrogenase (SDH) activity was assayed by incubating frozen sections in 0.1 M phosphate buffer, pH = 7.0, with 1.5 mM nitrotetrazolium blue chloride, 130 mM sodium succinate, 0.2 mM phenazine methosulfate, and 1 mM sodium azide for 10 min at 37 °C [65]. GPDH activity was assayed as an indirect measure of glycolytic activity; frozen sections were incubated in 0.1 M phosphate buffer, pH = 7.0, with 1.2 mM nitrotetrazolium blue chloride, 0.8 mM phenazine methosulfate, 9.3 mM glycerol phosphate disodium salt hydrate, and 1 mM sodium azide for 30 min at 37 °C [66]. Background staining was assessed in negative control slides by omitting enzyme substrates. Serial sections were used to determine enzyme activity in individual myofibers based on immunofluorescence labeling for MyHCs. Enzymatic activities of all myofibers on a whole section were performed by segmentation using Cellpose and quantification of optical density using ImageJ.

### 4.4. Immunoblot

Protein extraction and immunoblot assays were performed as conducted previously [61]. Muscle (50 µg) and kidney (5 µg) lysates were loaded alternating by genotype, subjected to SDS-PAGE, and proteins transferred onto nitrocellulose membranes. Equal protein loading was assessed using Ponceau S. After blocking membranes with 5% non-fat dry milk, membranes were incubated in anti-Klotho (1:500; #MABN1807, MilliporeSigma) in blocking solution and probed with HRP-conjugated anti-rat (#7077, Cell Signaling Technologies, Danvers, MA, USA) antibody. Bands were visualized using SuperSignal West Femto chemiluminescence substrate (ThermoScientific, Waltham, MA, USA) and captured using a Chemidoc Imager (BioRad, Hercules, CA, USA).

### 4.5. Serum Analyses

Creatine kinase (CK) activity (#MAK116, MilliporeSigma) and phosphate levels (#ab65622, Abcam, Cambridge, MA, USA) were measured in serum using commercially available assays. Absorbance was read using a SpectraMax M2 microplate reader (Molecular Probes, Eugene, OR, USA), and concentrations were calculated using regression analysis.

### 4.6. RNA Isolation, Quantitative Real-Time PCR (qRT-PCR), RNA-Sequencing, and Bioinformatic Analysis

RNA isolation and qRT-PCR assays were completed as performed previously [62]. Primers were designed in-house and commercially synthesized (MilliporeSigma). Primer specificity was verified by amplicon size using agarose gel electrophoresis and melt curve analysis for a single peak, and primer efficiency (90–105%) was assessed using serial dilutions of cDNA. Relative expression of selected transcripts (Supplementary Table S1) was normalized by geometric averaging the Cq values of 2 reference genes, *Ap3d1* and *Eef1a1*. The expression of each gene in control samples was set to one, and other expression values were then scaled to that value. RNA quality assessment, library construction, and mRNA sequencing were performed at the Indiana University School of Medicine Center for Medical Genomics. Briefly, 100 ng of RNA was used to generate libraries. Libraries were sequenced on a NovaSeq 6000 (Illumina, San Diego, CA, USA), generating approximately 40M reads per library. Quality control of raw sequence data was performed with FastQC (v0.11.5), and sequencing reads were mapped to the mm10 mouse reference genome using STAR (v2.7.10a). Low-quality mapped reads were excluded, featureCounts was used to quantify gene-level expression, and differential expression analysis was performed with edgeR. Gene Set Enrichment Analysis (GSEA) with Gene Ontology (GO) and Kyoto Encyclopedia of Genes and Genomes (KEGG) pathway functional enrichment analyses were performed using clusterProfiler.

### 4.7. In Vivo Plantarflexion Torque Assessment

Mice underwent in vivo plantarflexion torque assessment to assess contractility of the triceps surae (Aurora Scientific, Aurora, ON, CA). Briefly, mice were anesthetized with 2% isoflurane inhalation, and the right hindfoot was taped to the force transducer positioned at a 90° angle to the tibia, and the limb was fixed by a knee clamp, avoiding compression of the fibular nerve. Two disposable monopolar electrodes (Natus Neurology, Middleton, WI, USA) were inserted to stimulate the tibial nerve. Maximum twitch torque was first determined using supramaximal stimulations (0.2 ms square wave pulse). Peak plantarflexion torque was then assessed following a supramaximal square wave stimulation (0.2 ms) delivered at a frequency of 125 Hz. Peak tetanic torque and the maximum rate of force contraction and relaxation were analyzed using the Dynamic Muscle Control Data Analysis software (v5).

### 4.8. In Vivo Electrophysiology

Electrophysiological assessment was used to evaluate compound muscle action potential (CMAP) and MUNE as performed previously [67]. Mice were anesthetized with 2% isoflurane inhalation, and the sciatic nerve of the left limb was stimulated with two 28-gauge electrodes (Natus Neurology); a duo shielded ring electrode was used for recording, and a ground electrode was placed on the animal’s tail. Baseline-to-peak and peak-to-peak CMAP responses were recorded utilizing supramaximal stimulations (constant current intensity: <10 mA; pulse duration: 0.1 ms). Single motor unit potential (SMUP) size was determined using an incremental stimulation technique. Incremental responses were obtained by submaximal stimulation of the sciatic nerve until a stable, minimal all-or-none response occurred. Ten successive SMUP increments were recorded and averaged. MUNE was calculated by the following: CMAP amplitude (peak-to-peak)/average SMUP (peak-to-peak).

### 4.9. Statistics

All data are presented as mean ± s.e.m. Statistical significance was calculated by unpaired two-tailed Student’s *t*-test or two-way ANOVA with Holm–Šidák or Tukey’s multiple comparison test using GraphPad Prism (v10). Differences with *p* < 0.05 were considered statistically significant.

## Data Availability

The data presented in this study are available on reasonable request. RNA sequencing data have been deposited in the NCBI Gene Expression Omnibus (GEO) under accession number GSE302925.

## Supplementary Materials

The following supporting information can be downloaded at https://www.mdpi.com/article/10.3390/ijms26167986/s1.
