# Supplementary material for: Klotho Deficiency Promotes Skeletal Muscle Weakness and Is Associated with Impaired Motor Unit Connectivity
Source: Int J Mol Sci. 2025 Aug 19;26(16):7986. doi: 10.3390/ijms26167986 (PMC12386194; doi:10.3390/ijms26167986)
Supplement: Supplementary file 1 [file ijms-26-07986-s001.zip › ijms-3794628-SI.pdf]

Supplementary Table S1. qRTPCR Primers.

Gene	**Forward Primer**	**Reverse Primer**
Adgre1	5′-GATACAGCAATGCCAAGCAG-3′	5′-CAGCACGAGGGAGACACTT-3′
Ap3d1	5′-GATCATCAAGCTGTTCGGTGC-3′	5′-GAGATGAGCACAGCAATTACGG-3′
Ccr2	5′-CCTGTAAATGCCATGCAAGTTC-3′	5′-GTATGCCGTGGATGAACTGAG-3′
Chrna1	5′-GAGTGGGTGCGGAAGGTTT-3′	5′-GCGGAGAGTGAAAGCCCATA-3′
Chrna9	5′-CAGGTCACGCTCTCCCAGATA-3′	5′-GTACGCATCGTGCCAAGTTT-3′
Chrne	5′-GAAGCCACTGGAGAGGAACTG-3′	5′-TTGGATGCACGGTGGGTAAG-3′
Col5a3	5′-CGGGGTACTCCTGGTCCTAC-3′	5′-GCATCCCTACTTCCCCCTTG-3′
Cx3cr1	5′-CATGTGCAAGCTCACGACTG-3′	5′-CCCAGACGCCCAGACTAATG-3′
Eef1a1	5′-ACACGTAGATTCCGGCAAGTC-3′	5′-GATGGTTCGCTTGTCGATTCC-3′
Fn1	5′-GCTCAGCAAATCGTGCAGC-3′	5′-CTAGGTAGGTCCGTTCCCACTG-3′
Gdnf	5′-CCAGTGACTCCAATATGCCTG-3′	5′-CTCTGCGACCTTTCCCTCTG-3′
Itgam	5′-CATGAATGATGCTTACCTGGGTTATG-3′	5′-CCCAAAATAAGAGCCAATCTGG-3′
Kl	5′-GTCTCGGGAACCACCAAAAG-3′	5′-CTATGCCACTCGAAACCGTC-3′
Mstn	5′-GCACTGGTATTTGGCAGAGTA-3′	5′-GCAGTCAAGCCCAAAGTCTC-3′
Myh3	5′-GCAGACTGTGGACCAGGTTC-3′	5′-GCTGCTCCAGGCTGTTATACTC-3′
Myh4	5′-GCTTGAAAACGAGGTGGAAA-3′	5′-CCTCCTCAGCCTGTCTCTTG-3′
Myh6	5′-GCCCAGTACCTCCGAAAGTC-3′	5′-GCCTTAACATACTCCTCCTTGTC-3′
Myh8	5′-AACAGAAACGCAATGCTGAGG-3′	5′-TCGCCTGTAATTTGTCCACCA-3′
Myl4	5′-GGGCACCTATGAGGACTTCG-3′	5′-GGGTAGCAAGGACATGCCG-3′
Tnnt2	5′-GAGCTACAGACTCTGATCGAGG-3′	5′-TCCTTCTCCCGCTCATTGC-3′
